# Investigating the Correlation between Force Output, Strains, and Pressure for Active Skeletal Muscle Contractions.

**Published:** 2023-10-09

**Authors:** Karan Taneja, Xiaolong He, John Hodgson, Usha Sinha, Shantanu Sinha, J. S. Chen

**Affiliations:** a Department of Structural Engineering, University of California San Diego, San Diego, CA, USA; b ANSYS Inc, Livermore, CA, USA; c Department of Integrative Biology and Physiology, University of California Los Angeles, Los Angeles, CA, USA; d Department of Physics, San Diego State University, CA, USA; e Department of Radiology, University of California San Diego, San Diego, CA, USA

**Keywords:** skeletal muscle, force output, principal strain, volumetric strain, pressure, correlation

## Abstract

Experimental observations suggest that the force output of the skeletal muscle tissue can be correlated to the intra-muscular pressure generated by the muscle belly. However, pressure often proves difficult to measure through *in-vivo* tests. Simulations on the other hand, offer a tool to model muscle contractions and analyze the relationship between muscle force generation and deformations as well as pressure outputs, enabling us to gain insight into correlations among experimentally measurable quantities such as principal and volumetric strains, and the force output. In this work, a correlation study is performed using Pearson’s and Spearman’s correlation coefficients on the force output of the skeletal muscle, the principal and volumetric strains experienced by the muscle and the pressure developed within the muscle belly as the muscle tissue undergoes isometric contractions due to varying activation profiles. The study reveals strong correlations between force output and the strains at all locations of the belly, irrespective of the type of activation profile used. This observation enables estimation on the contribution of various muscle groups to the total force by the experimentally measurable principal and volumetric strains in the muscle belly. It is also observed that pressure does not correlate well with force output due to stress relaxation near the boundary of muscle belly.

## 1 Introduction

The role of simulations to study physiologically fruitful outcomes such as the effect of the passive material properties [1] and the effect of ageing [2] on skeletal muscle force generation is significant. Comparisons of simulation results with experimental data provide evaluation and potential validation of simulation work. A useful recent addition to experimental data has been the use of MRI in human subjects to provide higher resolution data on intramuscular deformation during muscle activity [3]. One challenge to such experimental validations in human subjects is that individual muscles generally cannot be isolated for physiological study since multiple muscles operate over a single joint. Currently we are unable to accurately determine the contribution of each muscle to the overall joint torque and thus, cannot directly relate stress and strain in the mechanical performance of individual muscles acting across a joint. The best that can currently be accomplished is to use surrogate measures of stress and express our measures relative to a maximum voluntary contraction. The most common measures use electromyograms (EMG) or intramuscular pressure (IMP) with varying degrees of confidence in their translation to stress among investigator groups [3–5]. The least certainty arises in dynamic contractions where sensor movement and velocity effects [6] may contaminate the recordings. Unfortunately, many of the MRI protocols require such dynamic contractions. Furthermore, IMP measures are (minimally) invasive which adds some risk and may deter some potential subjects. We were thus motivated to explore other surrogates of muscle force that could be conveniently applied in an MRI environment. Information on muscle-specific force contributions would have great clinical significance, advance diagnostic accuracy, and aid in the development of targeted therapies for a wide range of musculoskeletal pathologies.

One experimental measure of muscle function that has been shown to correlate with muscle force is the intra-muscular pressure (IMP). IMP is generated within an individual muscle and is a measure of the fluid pressure in a localized region and is a direct measure of the mechanical state of a muscle. Ex-vivo studies of isolated anterior tibialis muscle have shown that IMP is linearly related to muscle stress [5,7,8]. More recently, using a minimally invasive approach based on fiber optic sensors, Ates et al. [4] have shown that IMP identifies active mechanical properties of muscle in vivo and can be used to detect muscular changes due to drugs, diseases, or aging. This same group has shown that IMP measurement is an indicator of muscle performance in older adults [9]. These experimental studies have convincingly demonstrated that IMP is a surrogate measure of force and that individual muscle IMP and thus force can be measured *in-vivo*. However, it should be noted that the IMP is still an invasive procedure (albeit minimally so), and a completely non-invasive method would be preferable for studying normal and diseased muscle functions.

A number of computational modeling-based studies have explored the IMP-force relationship. Bojairami and Driscoll [10] correlated IMP and force via a three-dimensional finite element model by considering a neo-Hookean strain energy function for the skeletal muscle. Wheatley et al. [11] explored fluid content within the muscle tissue to predict force and IMP. This model was validated to predict both muscle stress and IMP under passive conditions for the New Zealand White Rabbittibialis anterior. Even though IMP has shown a correlation to muscle force in vivo, the clinical application of this technique is hindered by patient to patient and muscle to muscle variability resulting in the difficulty of utilizing IMP to estimate muscle force in the clinical applications. The investigation on mechanisms of IMP variability [12] found that fluid pressure was affected by muscle length under isometric conditions due to Poisson effects varying spatially, with the highest gradients located near aponeuroses. These factors were partially responsible for the variability in the IMP.

In an effort to explore the variability of IMP between subjects and muscles, Jensen et al. [13, 14] studied volumetric strain using sequentially acquired slices based on velocity encoded phase contrast imaging. The measurement of volumetric strain was motivated by the fact that the IMP and volumetric strain distributions may be related. With this hypothesis, the measured deformation under passive contraction showed regional variation of volumetric strain that had an inverse spatial variation to that of IMP [14]. Another focus of these studies was also to identify relatively homogeneous areas of volumetric strain which may potentially be areas for IMP sensor placement to ensure less variability among subjects. There have been recent advances in compressed sensing accelerated flow MRI that enables three-directional velocity imaging (3Dal) to allow the measurement of 3×3 strain tensors. The MRI measurements are completely non-invasive while in contrast, IMP measurements still require invasive placement of sensors. However, the correlation of volumetric strain to either IMP or force has not been established. A computational model is required to examine the relationship between IMP, volumetric and principal strains, and individual muscle force output. Establishing these correlations will enable experimentally observable deformation indices (e.g., volumetric strain) to be used as surrogate markers of individual muscle force. Further, such a correlation study among these quantities at different spatial locations of themuscle will better inform physiologists e.g., for regions of interest placement on imaging studies as well as for accurate placement of sensors to estimate intra-muscular pressure.

Viscoelastic effects play an integral role in the development of the intra-muscular pressure during muscle contraction over time, resulting from the active and passive responses of a 3-D composite material of muscle fibers and the extracellular matrix (MT) [15,16], inducing damping in the muscle contractions. It is thus important to include its contribution to the deformation and force output of the muscle. Numerical simulations have been employed to investigate the behaviors and mechanisms of skeletal muscle tissues. Starting from initial simplistic one-dimensional lumped parameter models [17], multi-dimensional continuum models have been favored by researchers in recent times [1,2,18,19]. The anisotropic properties of skeletal muscle have been simulated using transversely isotropic hyperelastic material models [1,2,18] with anisotropy introduced, e.g., through modeling the effect of collagen fibers wrapped around the muscle fiber [1]. However, these models do not account for the viscous effects observed for skeletal muscle tissues in experiments [16]. To generate more physiologically realistic computational models, researchers have included visco-hyperelasticity to model skeletal muscle behavior [15,20,21]. In this study, we use a visco-hyperelastic framework to simulate isometric contractions in a skeletal muscle to identify the correlations between the force output of the entire model and the pressure and various strain measures calculated at different locations in the muscle, for linear and non-linear activation profiles.

This paper describes our initial steps to develop a framework to explore the relationship between intramuscular pressure, principal and volumetric strains, and force output. Through a systematic correlation study, this study allows one to derive meaningful conclusions on the relationship of observable deformation outputs, specifically the strains, and its influence on muscle force generation and individual muscle force contribution to the total force measured during contractions [4]. The remainder of this manuscript is organized as follows. Section 2 introduces the material models of various components in the continuum-scale muscle model. In Section 3, the responses of the continuum-scale skeletal muscle simulations for isometric contractions are investigated to study the influences of activations on muscle force output, pressure, and deformation. Finally, conclusion remarks are made in Section 4.

## 2 Methods

**Fig. 1:**
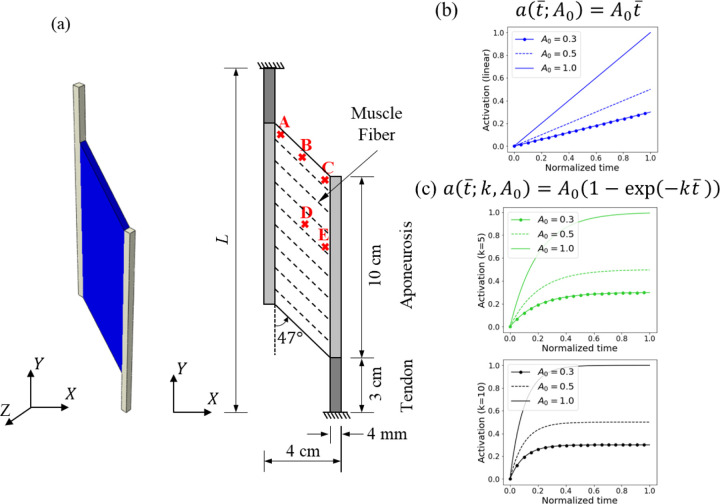
An overview of the proposed modeling of the skeletal muscle. The model in (a) is the 3D continuum-scale plane strain model with an initial pennation angle of 47° and a thickness of 0.4 cm in the z-direction, where the dash lines represent the orientation of muscle fibers. The five positions A→E in the belly indicate locations where the pressure and volumetric strain are extracted for the statistical analysis. The figures in (b) and (c) show the nine activation profiles described in Table 4. $\overset{‾}{t}$ is the normalized time.

A generic 3D continuum muscle model with initial (i.e., prior to fiber activation) pennation angle $\theta =47^{\circ }$ and a thickness of 0.4 cm is shown in Fig. 1(a). Fixed boundary conditions i.e., the top and bottom ends of the tendon are kept fixed to simulate isometric muscle contractions at different activation levels. The continuum-scale muscle components are modeled by a hybrid finite element formulation with a bilinear displacement field and a constant pressure field. The continuum muscle simulations are quasi-static under the plane strain condition, which is consistent with the experimental observations that the out-of-plane deformation is relatively small compared to the in-plane deformation [1,22].

### 2.1 Continuum Muscle Model

The continuum muscle-tendon complex (Fig. 1) contains muscle fibers, anisotropic matrix, aponeurosis, and tendon. The following subsections describe the employed material models and visco-hyperelastic effects.

#### 2.1.1 Hyperelastic Material Models for the Continuum Muscle Model

In the continuum muscle-tendon complex, the tendon and the aponeurosis are modeled by an isotropic third-order generalized Mooney-Rivlin model to represent softer responses in the low-strain region and stiffer responses in the high-strain region, given as

(1) $$W_{\text{tendon }}\left({\overset{‾}{I}}_{1},J\right)=\sum_{i=1}^{3}\;C_{i0}{\left({\overset{‾}{I}}_{1}-3\right)}^{i}+\frac{K}{2}(J-1{)}^{2}.$$

Herein, ${\overset{‾}{I}}_{1}=J^{-2/3}I_{1}$, are the reduced invariants of the right Cauchy-Green Tensor $C=F^{T}F$ with $I_{1}=\text{tr}(C),I_{2}=\frac{1}{2}\left[I_{1}^{2}-\text{tr}\left(C^{2}\right)\right],J=\text{det}(F),F=\frac{\partial x}{\partial X}$ is the deformation gradient, where x and X are the deformed and undeformed material coordinates. The material constants are given in Table 1.

As the continuum skeletal muscle contains both fiber and matrix components, the strain energy density function of the continuum skeletal muscle is decomposed into three parts, (i) a passive deviatoric matrix (MT) part, (ii) a passive volumetric MT part, and (iii) an anisotropic (contractile) part of muscle fibers (FB):

(2) $$W_{\text{muscle }}=W_{\text{MT}}^{\text{dev}}\left({\overset{‾}{I}}_{1},{\overset{‾}{I}}_{2},{\overset{‾}{I}}_{4}\right)+W_{\text{MT}}^{\text{vol}}(J)+W_{\text{FB}}^{\text{ani}}(\lambda ,\dot{\lambda }),$$

where the deviatoric part $W_{\text{MT}}^{\text{dev}}\left({\overset{‾}{I}}_{1},{\overset{‾}{I}}_{2}\right)$ is defined as a generalized Mooney-Rivlin model to describe the transversely isotropic passive material properties,

(3) $$W_{\text{MT}}^{\text{dev}}\left({\overset{‾}{I}}_{1},{\overset{‾}{I}}_{2},{\overset{‾}{I}}_{4}\right)=\sum_{i+j=1}^{3}\;C_{ij}{\left({\overset{‾}{I}}_{1}-3\right)}^{i}{\left({\overset{‾}{I}}_{2}-3\right)}^{j}+k_{0}\left\{\text{exp}\left[k_{1}{\left({\overset{‾}{I}}_{4}-1\right)}^{2}\right]-1|,\right\}$$

with ${\overset{‾}{I}}_{2}=J^{-4/3}I_{2}$ and an exponential term of stretch ratio associated with muscle fibers and collagen fibers along the fiber direction, ${\overset{‾}{I}}_{4}=J^{-2/3}N\cdot C\cdot N,N$ is the unit vector in the fiber direction and $C_{ij},k_{0}$, and $k_{1}$ are the material constants calibrated from the data obtained from the microstructure homogenization protocols described in [1] as shown in Table 2. The volumetric part $W_{\text{MT}}^{\text{vol}}(J)$ is used to represent nearly incompressible materials,

(4) $$W_{\text{MT}}^{\text{vol}}(J)=\frac{K}{2}(J-1{)}^{2}.$$

with the same bulk modulus $K=10^{5}\;\text{N}/{\text{cm}}^{2}$ used for $W_{MT}^{vol}$ and $W_{\text{tendon }}$.

**Fig. 2:**
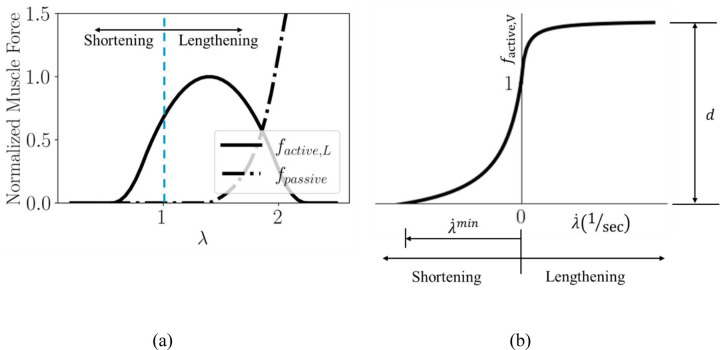
Normalized (a) length dependent, and (b) velocity dependent mechanical responses of the muscle fiber model with $a(\overset{‾}{t})=1$. The stretch andstretch rate at which the shortening and lengthening phases of the fiber exist are also indicated.

The anisotropic part $W_{FB}^{ani}$ is used to describe the contractile Cauchy stress in the fiber $\sigma_{FB}$, including the active-length dependent and velocity dependent effects as considered by [15,23,24],

(5) $$\sigma_{FB}=\lambda \frac{\partial W_{\text{FB}}^{ani}(\lambda ,\dot{\lambda })}{\partial \lambda }=\sigma_{max}\frac{\lambda }{\lambda_{0}}\left(a(\overset{‾}{t})f_{\text{active},L}f_{\text{active},V}+f_{\text{passive }}\right)$$

where $\sigma_{max}$ is the fiber maximum isometric stress, $\overset{‾}{t}$ is the normalized time for activation, λ is the fiber stretch, $\lambda_{0}=1.4$ is selected as the optimal along-fiber stretch ratio at which the muscle fiber generates maximum force. $f_{\text{active,L }}$ and $f_{\text{passive }}$ are the normalized active-length dependent and passive parts of the muscle fiber force, [1], respectively, expressed as,

(6) $$f_{\text{active},L}=\left\{\begin{array}{ll} 9{\left(\lambda^{*}-0.4\right)}^{2}, & \lambda^{*}\leq 0.6\\ 1-4{\left(1-\lambda^{*}\right)}^{2}, & 0.6<\lambda^{*}\leq 1.4\\ 9{\left(\lambda^{*}-1.6\right)}^{2}, & \lambda^{*}>1.4 \end{array}\right.$$

(7) $$f_{\text{passive }}=\left\{\begin{array}{ll} 0, & \lambda^{*}\leq 1\\ \gamma_{1}\left[\left(e^{\gamma_{2}\left(\lambda^{*}-1\right)}-1\right)\right], & 1<\lambda^{*}\leq 1.4\\ \left(\gamma_{1}\gamma_{2}e^{0.4\gamma_{2}}\right)\lambda^{*}+\gamma_{1}\left[\left(1-1.4\gamma_{2}\right)e^{0.4\gamma_{2}}-1\right], & \lambda^{*}>1.4 \end{array}\right.$$

where $\lambda^{*}$ is the normalized stretch as $\lambda^{*}=\lambda /\lambda_{0}$. The velocity dependent fiber force, $f_{\text{active, }V}$, is described as, [23],

(8) $$f_{\text{active},V}=\left\{\begin{array}{ll} \frac{1-\left(\frac{\dot{\lambda }}{{\dot{\lambda }}^{\text{min }}}\right)}{1+k_{c}\left(\frac{\dot{\lambda }}{{\dot{\lambda }}^{\text{min }}}\right)}, & \dot{\lambda }\leq 0\\ d-(d-1)\frac{1+\left(\frac{\dot{\lambda }}{{\dot{\lambda }}^{\text{min }}}\right)}{1-k_{c}k_{e}\left(\frac{\dot{\lambda }}{{\dot{\lambda }}^{\text{min }}}\right)}, & \dot{\lambda }>0 \end{array}\right.$$

where ${\dot{\lambda }}_{\text{min }}$ is the minimum stretch rate of the fiber. The first equation in Eq. (8) describes the concentric phase, where $k_{c}$ is a dimensionless constant controlling the curvature of the force vs fiber contraction velocity plot. The second equation describes the eccentric phase, where the muscle develops tension as it lengthens, and another dimensionless constant $k_{e}$ describes the curvature in this phase. The dimensionless constant d is the offset of the eccentric function. Fig. 2 shows both length and velocity dependent force production from the muscle fiber.

#### 2.1.2 Visco-Hyperelastic Formulation

The viscous effects are only included for the muscle belly, excluding the tendons and aponeurosis. We begin with the hyperelastic formulation of the 2nd Piola-Kirchhoff (PK) stresses which can be decomposed into deviatoric, volumetric and muscle fiber stresses.


(9) $${\tilde{S}}_{ij}={\tilde{S}}_{ij}^{dev}+{\tilde{S}}_{ij}^{vol}+{\tilde{S}}_{ij}^{FB}$$

The deviatoric and volumetric $2^{\text{nd }}$ PK stresses are written as

(10) $${\tilde{S}}_{ij}^{dev}=\frac{\partial W_{MT}^{dev}\left({\overset{‾}{I}}_{1},{\overset{‾}{I}}_{2},{\overset{‾}{I}}_{4}\right)}{\partial E_{ij}},{\tilde{S}}_{ij}^{vol}=\frac{\partial W_{MT}^{vol}(J)}{\partial E_{ij}}$$

where the superscript ‘~’ denotes hyperelastic stresses, and $E_{ij}=\frac{1}{2}\left(C_{ij}-\delta_{ij}\right)=\frac{1}{2}\left(F_{ki}F_{kj}-\right.{\;\delta }_{ij})$ is the Lagrangian strain tensor. The fiber contractile Cauchy stress in Eq. (5) can be transformed to the 2^nd^ PK stress ${\tilde{S}}_{ij}^{FB}$. For fiber 2nd PK stress, this can be simplified by dividing the fiber Cauchy stress in Eq. (5) by the stretch ratio in the fiber direction, and then rotate it to the Cartesian coordinate in the undeformed configuration.

The viscous behaviour is introduced into the deviatoric and volumetric components of muscle matrix using the continuous generalized Maxwell formulation [25,26] (see Appendix A for details), and the final expressions are as follows.

(11) $$S_{ij}^{dev}\left(t_{n+1}\right)\approx S_{ij,n+1}^{dev}={\tilde{S}}_{ij,n+1}^{dev}+\sum_{p=1}^{N}\;\;{\overset{‾}{g}}_{p}H_{p,ij}^{n+1}$$

(12) $$S_{ij,n+1}^{vol}={\tilde{S}}_{ij,n+1}^{vol}+\sum_{p=1}^{N}\;\;{\overset{‾}{b}}_{p}L_{p,ij}^{n+1}$$

(13) $$H_{p,ij}^{n+1}=exp\left(-\frac{\Delta t}{\tau_{p}}\right)H_{p,ij}^{n}+\left(\frac{1-exp\left(-\frac{\Delta t}{\tau_{p}}\right)}{\frac{\Delta t}{\tau_{p}}}\right)\left({\tilde{S}}_{n+1,ij}^{dev}-{\tilde{S}}_{n,ij}^{dev}\right)$$

(14) $$L_{p,ij}^{n+1}=exp\left(-\frac{\Delta t}{\tau_{p}}\right)L_{p,ij}^{n}+\left(\frac{1-exp\left(-\frac{\Delta t}{\tau_{p}}\right)}{\frac{\Delta t}{\tau_{p}}}\right)\left({\tilde{S}}_{n+1,ij}^{vol}-{\tilde{S}}_{n,ij}^{vol}\right).$$

where ${\overset{‾}{g}}_{p}$ and ${\overset{‾}{b}}_{p}(p=1\ldots N)$ are the N-term Prony-series deviatoric and volumetric relaxation coefficients, respectively, given in Table 3, and the derivation of $H_{p,ij}^{n+1}$ and $L_{p,ij}^{n+1}$ are given in Appendix A. Both deviatoric and volumetric Prony series use the same coefficients in our model. The total 2^nd^ PK stress at time step n+1 is then written as

(15) $$S_{n+1,ij}=S_{n+1,ij}^{dev}+S_{n+1,ij}^{vol}+{\tilde{S}}_{n+1,ij}^{FB}$$

The tangent obtained due to the linearization process also needs to be updated at each time step.


(16) $${𝒞}_{\text{ijkl}}^{n+1}=\frac{\partial S_{n+1,ij}}{\partial E_{kl}^{n+1}}=\frac{\partial S_{n+1,ij}^{dev}}{\partial E_{kl}^{n+1}}+\frac{\partial S_{n+1,ij}^{vol}}{\partial E_{kl}^{n+1}}+\frac{\partial {\tilde{S}}_{n+1,ij}^{FB}}{\partial E_{kl}^{n+1}}$$

The expressions of the tangent matrix components are given in Appendix B. Using the stress and constitutive tensor updates, one can perform a nonlinear analysis.

## 3 Results and Discussions

### 3.1 Numerical Setup

The continuum-scale model under isometric contractions is simulated as described in Section 2 and shown in Fig. 1. The muscle belly undergoes nine different activations (L1 – 3, NL1 – 6), which are described in Table 4 and shown in Fig. 1(b)-(c).

For the correlation study discussed in Section 3.3, the quantities under investigation are extracted from five locations (A,B,C,D,E) on the muscle belly as shown in Fig. 1(a). The force output is measured as the reaction of the model at the bottom support. The pressure observed in the muscle belly at the initial (t=0.1sec), middle (t=0.5sec) and final (t=1sec) stages of the isometric contraction is shown in Section 3.2, and is defined as

(17) $$p=-\frac{tr(\sigma )}{3}$$

where σ is the Cauchy stress.

The volumetric strain $\epsilon_{vol}$ observed in the muscle belly is defined as

(18) $$\epsilon_{vol}=tr\left(\epsilon^{N}\right)$$

where $\epsilon^{N}=\sqrt{F\cdot F^{T}}-I$ is the nominal strain, and F is the deformation gradient. The principal strains are calculated from the nominal strains, where the maximum and minimum principal strains are denoted as $\epsilon_{1}$ and $\epsilon_{2}$, respectively. The maximum shear strain is then obtained as

(19) $$\gamma_{\text{max}}=\frac{\epsilon_{1}-\epsilon_{2}}{2}.$$

### 3.2 Simulation Results

#### Force Output

**Fig. 3:**
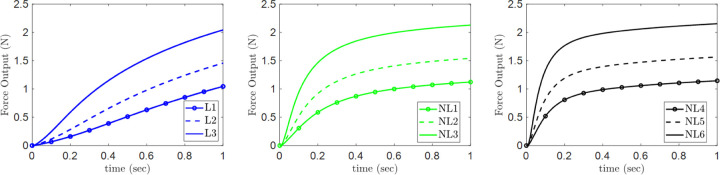
Force output from the skeletal muscle model vs time for the linear (L1-L3) and non-linear (NL1-NL6) activation profiles.

The force output closely follows a similar activation profile as evident from Fig. 3. They all reach similar levels of peak force output measured at the end of the simulation, corresponding to the maximum activation of the muscle belly, $A_{0}$. For the cases with non-linear activation, the rise to the peak force is quicker than their linear counterparts, but they arrive at their peak force values.

#### Pressure

An increase in the pressure of the belly was observed as the activation increases; more pronounced in the case of a higher activation level $\left(A_{0}=1.0\right)$. The results show the strongest correlation between the maximum activation level and the maximum pressure at 100% activation $\left(A_{0}=1.0\right)$ followed by 50% $\left(A_{0}=0.5\right)$ and then 30% $\left(A_{0}=0.3\right)$, for each of the linear and nonlinear profiles. The lowest pressure is observed along the traction-free surfaces at the top and bottom of the muscle belly.

#### Strain Measures

The maximum principal strain $\left(\epsilon_{1}\right)$ along with the maximum shear strain $\left(\gamma_{max}\right)$ and volumetric $\left(\epsilon_{vol}\right)$ strain are considered for their correlation to muscle activation. The $\epsilon_{vol}$ is discussed here whereas the distributions of $\epsilon_{1}$ and $\gamma_{max}$ are shown in Appendix C, as they show a similar relationship with the muscle activation.

The $\epsilon_{vol}$ increases throughout the belly as the activation increases. The maximum $\epsilon_{vol}$ observed shows a strong correlation to the maximum activation level, with a majority of the belly undergoing an increase in the $\epsilon_{vol}$ as the contraction progresses to its final state.

### 3.3 Correlations between Force Output, Pressure and Strain Measures

To analyze the relationship between the variables of interest, the evolution of the force output, pressure and principal and volumetric strains, at various activation levels and profiles, were plotted pairwise with respect to each other in Fig. 6 - Fig. 8. On each curve, the markers are plotted with every five data-points from a total of one hundred data-points.

For linear activation profiles, we observe strong positive correlations between force output, pressure, and various strain measures. For non-linear activations, the volumetric strain, maximum principal strain and maximum shear strain vs force showed a similar positive correlation. A positive correlation indicates that as the variable on the x-axis increases, so does the variable on the y-axis. The volumetric strain vs pressure and pressure vs force relations, however, show non-monotonic relationships at sampling locations away from the muscle belly center. It is also observed that these quantities at locations D and E show monotonicity; as pressure increases, so does volumetric strain, and as force increases, so does the pressure, maximum principal, maximum shear, and volumetric strain. This is not the case at locations A, B and C for pairwise plots with pressure as a variable, as they are away from the center of the belly, on the traction free surface. These results can be explained by the presence of stress relaxation at locations A, B and C, where the pressure decreases after the activation reaches its peak due to stress relaxation and the pressure reduction trend continues for the rest of the isometric contraction where the activation stays at the peak. As the activation profiles are such that the magnitude does not change after reaching peak activation $\left(A_{0}\right)$, the faster the rise to $A_{0}$, the more time for the stress relaxation near the traction free surface to occur. It is noted that the force output and maximum principal and maximum shear strains show very strong linear monotonic correlations for each activation magnitude $A_{0}$. These correlations will be further quantified in the following sub-sections.

Given the apparent relations observed from the simulations between the force output, pressure, and the principal and volumetric strains, at various activation levels, correlation analysis was conducted to investigate linear and monotonic relationships between these variables. A rudimentary test for correlation between two variables X and Y is to measure their linear correlation by the Pearson’s correlation coefficient, defined as

(20) $$r_{p}(X,Y)=\frac{\text{cov}(X,Y)}{\sqrt{\text{var}(X)}\sqrt{\text{var}(Y)}}$$

where X and Y are the random variables between which the correlation is measured.

However, variables may be correlated non-monotonically, as can be seen for the pressure vs volumetric strain and force vs volumetric strain plots in Fig. 6, Fig. 7 and Fig. 8. For such a situation, the variables may move in the same/opposite direction at variable rates whereas the rate is constant in a linear correlation. The Spearman’s coefficient can be used to measure the monotonic correlation between a pair of random variables X and Y, defined as

(21) $$r_{s}(X,Y)=\frac{\text{cov}(R(X),R(Y))}{\sqrt{\text{var}(R(X))}\sqrt{\text{var}(R(Y))}}$$

The variables X and Y are first converted to their ranked variables $R\left(x_{i}\right),R\left(y_{i}\right)$ such that they are ranked according to the magnitude of the $i^{\text{th }}$ samples, $x_{i}\in X$ and $y_{i}\in Y$. The correlations are then measured between the ranked variables R(X) and R(Y). The Spearman’s coefficient ranges from −1 to +1, where $r_{s}=\pm 1$ indicates a perfectly monotonically increasing/decreasing relationship between X and Y. For a monotonically increasing relationship, as X increases, Y also increases but the correlation could be linear or higher order. Likewise, in a monotonically decreasing correlation, as one variable increases, the other one decreases.

#### 3.3.1 Linear correlations

The Pearson’s correlation coefficient was calculated at five locations (A→E) indicated in Fig. 1. Fig. 9 shows the resultant correlations between force output, pressure, and maximum principal, maximum shear, and volumetric strains at the five locations. As observed from the pairwise plots in Fig. 6 - Fig. 8, strong positive linear correlations were observed between the pairs of the six quantities at locations D and E. Locations A, B and C, which are on the traction free surface, still show strong positive linear correlations between the force and principal strain quantities. However, they also show a drop in pressure as the force and volumetric strain increase, indicating a weak correlation leading to lower Pearson’s coefficient between them as a whole. It should be noted that as the magnitude of activation $\left(A_{0}\right)$ increases, the local non-monotonicity in the force vs pressure and pressure vs volumetric strain plots amplifies towards the end of the contraction as shown in Fig. 6 - Fig. 8, while their Pearson’s correlation remain strong at all locations (Fig. 9). This indicates that the Pearson’s coefficient is unable to reflect the existence of the local non-monotonicity. For this reason, other methods such as the Spearman’s coefficients were calculated and discussed in Section 3.3.2.

Interestingly, linear activation profiles for all three maximal activations lead to strong co-dependence of these quantities. This was shown in Fig. 6 - Fig. 8 where for linear activation profiles, the correlation is strongly linear. Whereas for non-linear activations, the pressure-volumetric strain and force-pressure plots show a weaker linearity and local non-monotonicity. In such conditions, Pearson’s correlation measure is less representative, and Spearman’s offers a better alternative as discussed in the next sub-section.

#### 3.3.2 Monotonic correlations

As described before, Pearson’s correlation coefficient does not capture the local non-monotonicity, reflecting only the global linear correlation instead. For this, the Spearman’s correlation coefficient was calculated at five locations (A→E) as shown in Fig. 10.

In Fig. 10, a strong monotonically increasing relationship is observed between the pairs of these quantities at location D, except for the case of k=10, where the nonlinear activation rises to peak the fastest. All other locations show varying degrees of weak monotonicity between (pressure volumetric strain) and (force, pressure), leading to a lower $r_{s}$ coefficient at location E and $r_{s}\approx 0$ at locations A, B and C. The monotonic relationship in the force vs maximum principal strain and maximum shear strain, shown in Fig. 6 - Fig. 8 shows a strong positive correlation for all cases and locations in Fig. 10.

It is also worth noting that the majority of the data-points are with local non-monotonic correlation for contractions with non-linear activations (k=5,10) and higher activation magnitude $\left(A_{0}=0.5,1.0\right)$, which the Pearson’s coefficient fails to account for. The Spearman’s coefficient used to quantify monotonicity offers a more accurate measure for variables with local non-monotonic correlations. Most noticeably, a strong monotonic correlation exists between force and all strain measures considered at all locations and under all activation functions as demonstrated in Fig. 10.

In summary, these results indicate that as force increases during the contraction, the maximum principal, maximum shear and volumetric strain increases whereas minimum principal strain decreases monotonically at all locations, indicated by both the strong Pearson’s and Spearman’s correlation coefficients as shown in Fig. 9 and Fig. 10. On the contrary, force-pressure and pressure-volumetric strain do not correlate well at the traction free surface and at the location near the boundary between the muscle belly and aponeurosis due to stress relaxation. It is, however, evident that the location at the center of the belly shows the strongest correlation between the force, pressure, and principal and volumetric strains, indicating a potentially best location to correlate these quantities. These results, which are also consistent with those observed by other numerical and experimental studies [5,10], have a major implication on the experimental design and sensor placement for IMP measurements.

## 4 Conclusions

MRI imaging to experimentally measure intramuscular deformation requires dynamic contractions rather than static images at different levels of contraction [3]. Furthermore, it would be highly instructive to correlate forces with strain, requiring measures of muscle force output. This presents a challenge when minimally invasive procedures are desirable. Electromyograms (EMG) and intramuscular pressure (IMP) have been used as surrogates for muscle forces expressed as a fraction of a maximal voluntary contraction (MVC) [5,27]. The relationships between muscle force and EMG or IMP remain somewhat controversial, may be non-linear, change with joint angle and require calibrations which may be complicated by changing contributions from multiple muscles contributing to joint torque usually used as a surrogate for muscle force [4,9]. Furthermore, there is general agreement that the utility of these measures deteriorates in dynamic contractions [6]. In an effort to explore alternative, more reliable proxies for muscle force, we used computer simulations to investigate the relationship between various strain measures and force output, a potential alternative that is non-invasive and easy to apply in the MRI environment [13,14].

In this study, a visco-hyperelastic modeling approach has been applied to continuum-scale skeletal muscle modeling to investigate the correlations between the force output, various strain measures (volumetric, maximum principal and maximum shear strains), and the pressure of the continuum skeletal muscle. While strains such as the volumetric strain and force at the joint can be measured *in-vivo* and non-invasively, important quantities such as the pressure experienced by the muscle belly during contractions can only be monitored by sensors that are embedded invasively. On the other hand, a numerical simulation and correlation analysis on the three quantities offer alternatives to investigate the dependence of pressure and volumetric strain on the force output. The numerical investigation of such a relationship establishes volumetric strain as highly correlated to force and future experimental studies can use the volumetric strain as a measure of single muscle force.

**Fig. 4:**
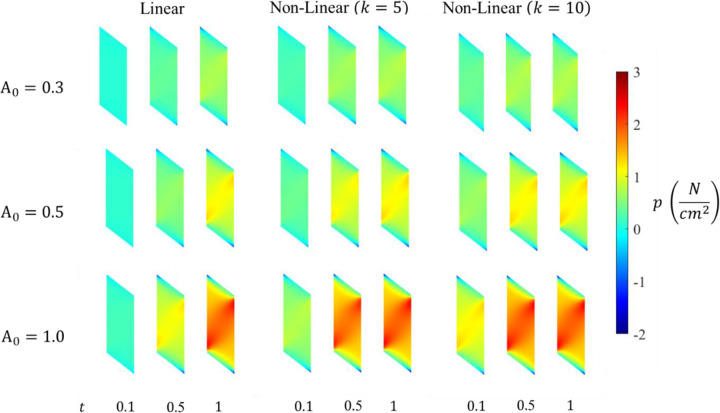
Pressure distributions at three different time steps for varying activation profiles and maximum activation levels $\left(A_{0}\right)$ in the muscle belly.

**Fig. 5:**
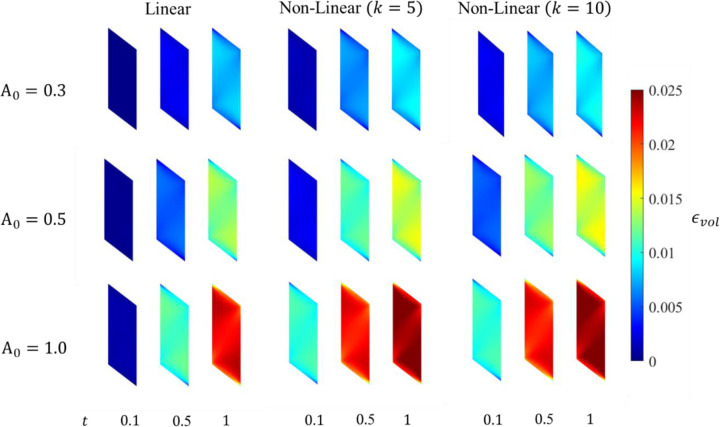
Volumetric strain distributions at three different time steps for varying activation profiles and maximum activation levels $\left(A_{0}\right)$ in the muscle belly.

**Fig. 6:**
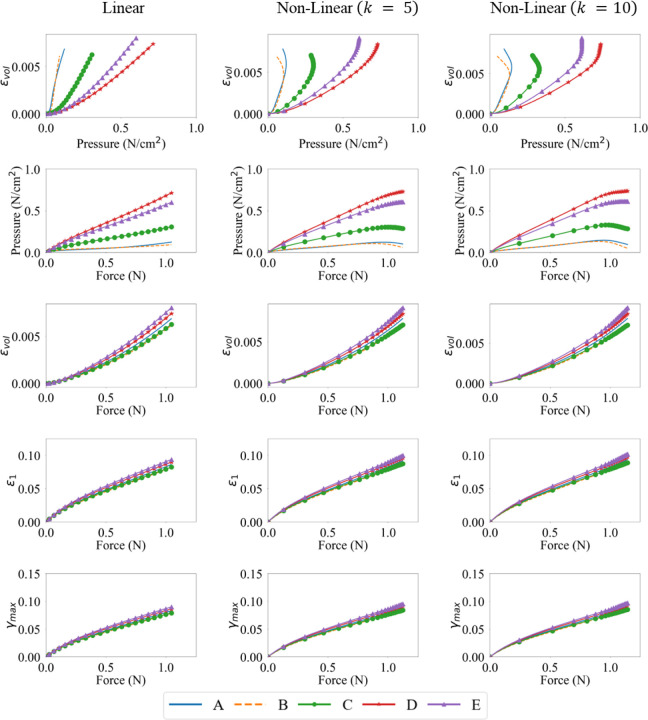
Evolution of the force output, pressure and maximum principal, maximum shear and volumteric strains for linear and non-linear activation profiles, for a maximal activation of $A_{0}=0.3$, at locations A-E on the muscle belly.

**Fig. 7:**
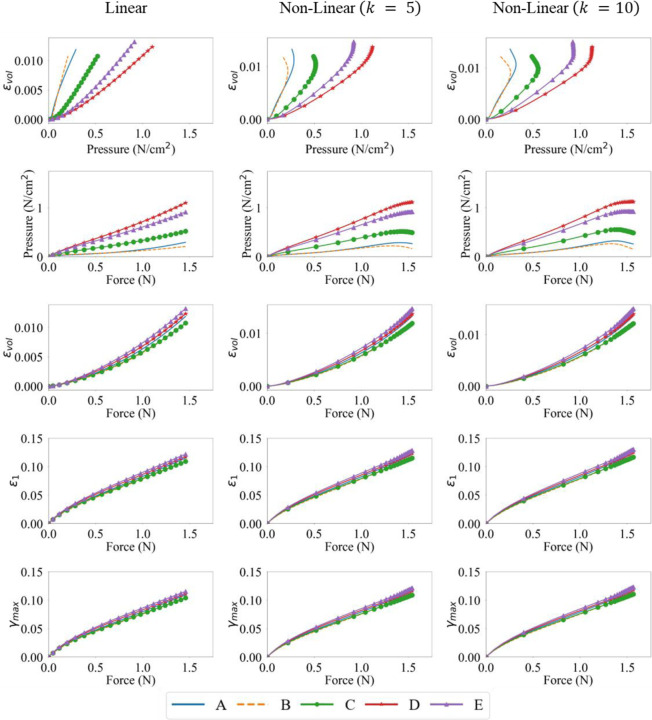
Evolution of the force output, pressure and maximum principal, maximum shear and volumteric strains for linear and non-linear activation profiles, for a maximal activation of $A_{0}=0.5$, at locations A-E on the muscle belly.

**Fig. 8:**
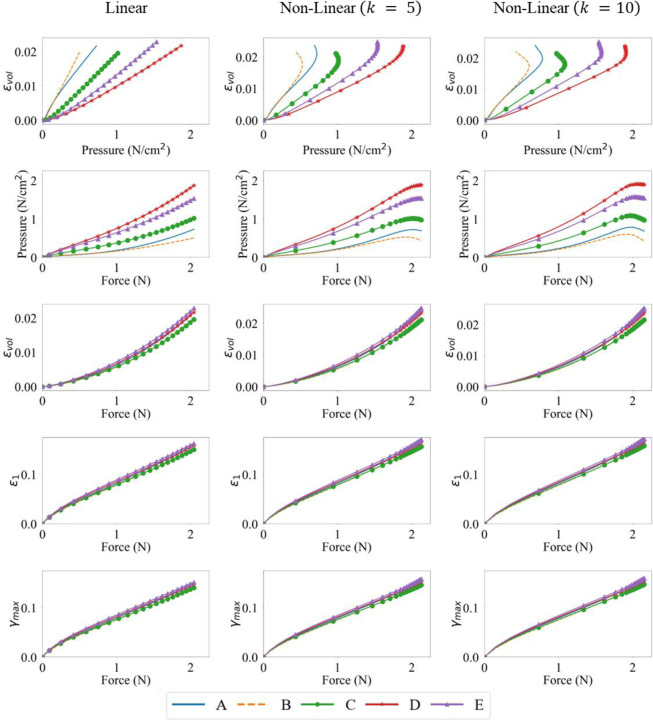
Evolution of the force output, pressure and maximum principal, maximum shear and volumteric strains for linear and non-linear activation profiles, for a maximal activation of $A_{0}=1.0$, at locations A-E on the muscle belly.

**Fig. 9:**
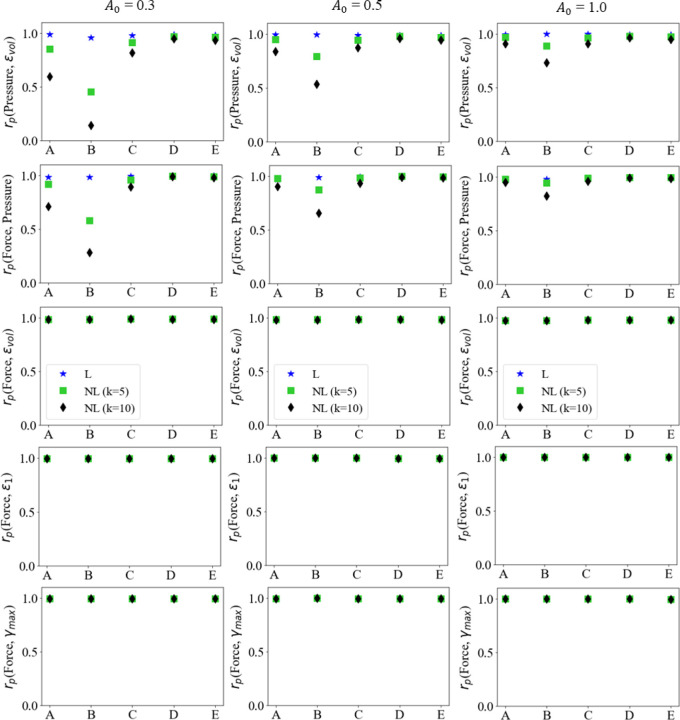
Pearson’s correlation coefficient plots between Force output, Pressure and Volumetric, Maximum Principal, and Maximum Shear Strains at five locations in the muscle belly.

**Fig. 10:**
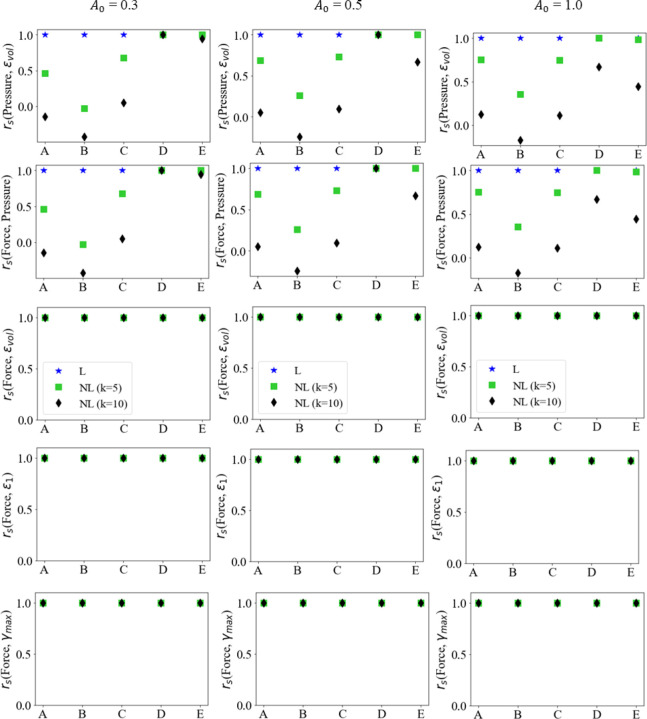
Spearman’s correlation coefficient plots between Force output, Pressure and Volumetric, Maximum Principal, and Maximum Shear Strains at five locations in the muscle belly.

The continuum-scale skeletal muscle model is subjected to isometric contraction with linear and non-linear activation profiles. The distribution patterns of pressure and volumetric strain appear qualitatively similar (Fig. 4 and Fig. 5). However, the magnitudes of the changes are reversed. Pressure in the contracted muscle, decreases proximo-distally and also towards the myotendinous junctions whereas the volumetric strain is lower in the center of the muscle and generally increasing towards the aponeuroses, although small proximal and distal regions of the muscle exhibit the lowest volumetric strains. It is also clear from these figures and from the separation of the regional plots in in Fig. 6 - Fig. 8 that the variation of volumetric strain across the muscle is about 20% whereas pressure varies by more than 100% across the muscle, suggesting that volumetric strain may be less susceptible to changes in sample location. The very limited reports of experimental measures of volumetric strain only discuss changes during passive movement of the human Tibialis anterior muscle but suggest a much higher variability across the muscle with absolute strains an order of magnitude higher than our simulations predicted [13,14]. We have currently not investigated simulations during passive movements, nor have we included potential vascular effects in this initial model.

Linear (Pearson’s correlation) and Monotonic (Spearman’s correlation) correlation measures were employed to investigate the correlations between the pressure, the principal and volumetric strain measures, and the force output. It is observed that the strongest correlations between these variables happen at the center of the belly for both linear and non-linear activations. It is also observed that Spearman’s correlation provides a better correlation measure associated with the local non-monotonicity. A strong correlation exists between all the strain measures (maximum principal strain, maximum shear strain and volumetric strain) and force output at all locations of the belly, irrespective of the activation profiles. Since strains are experimentally measurable, e.g., through MRI, this observation provides a pathway for better estimating the force in an individual muscle relative to its maximum effort, by using the experimentally measurable principal strains and volumetric strain in the muscle belly. It is also observed that pressure does not correlate well with force output due to stress relaxation near the boundary of muscle belly.

Whilst our results show some promise for volumetric strain as a proxy of force, experimental measures demonstrate the feasibility of measuring volumetric strain from MRI data but are not entirely consistent with the results of our simulations. Volumetric strain was measured in the human Tibialis Anterior muscle during passive ankle rotations [13,14]. In general, strains were an order of magnitude greater in the experimental studies, possibly because our simulation did not incorporate the potential influence of changes in intramuscular blood volume on volumetric strain.

In future studies, continuum-scale muscle models constructed from MRI scans of real muscles will be incorporated into this study for more realistic geometry effects on these measures and their correlations. More realistic activation profiles such as a quarter-sinusoidal function will also be tested. These studies will also be conducted on muscle lengthening and shortening to obtain a complete picture of the contractile behaviors of the fiber.

## Figures and Tables

**Fig. 11:**
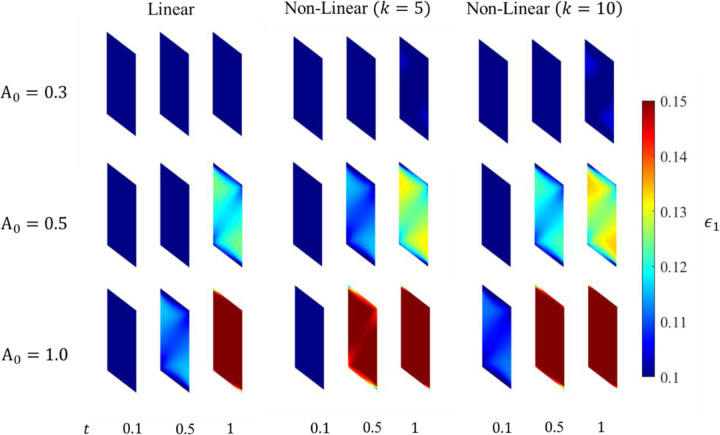
Maximum principal strain evolution in the muscle belly at three different activation times.

**Fig. 12:**
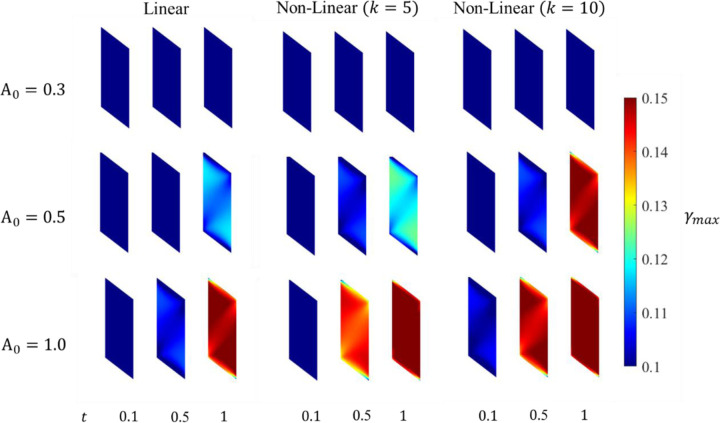
Maximum shear strain evolution in the muscle belly at three different activation times.

**Table 1:** Material parameters of $W_{\text{tendon }}$ in Eq. (1) for tendon and aponeurosis (unit: $N/{cm}^{2}$) [1].

$C_{10}$	$C_{20}$	$C_{30}$	K
30	80	800	$2\times 10^{5}$

**Table 2:** Material parameters of $W_{MT}^{\text{dev}}$ in Eq. (3) (unit: $N/{cm}^{2}$).

$C_{-10}$	$C_{01}$	$C_{20}$	$C_{11}$	$C_{02}$	$C_{30}$	$C_{21}$	$C_{12}$	$C_{03}$	$k_{0}$	$k_{1}$
2.23	−1.03	−10.89	24.13	−4.89	10.65	−16.36	8.21	−1.30	0.58	0.69

**Table 3:** The deviatoric (and volumetric) relaxation and time coefficients from the five term Prony series for muscle tissue used in [16].

Relaxation Coefficients	Value	Time Coefficients	Value(sec)
${\bar{g}}_{1}\left(={\bar{b}}_{1}\right)$	3.780487805	τ1	0.6
${\bar{g}}_{2}\left(={\bar{b}}_{2}\right)$	1.62601626	τ2	6
${\bar{g}}_{3}\left(={\bar{b}}_{3}\right)$	0.463414634	τ3	30
${\bar{g}}_{4}\left(={\bar{b}}_{4}\right)$	0.536585366	τ4	60
${\bar{g}}_{5}\left(={\bar{b}}_{5}\right)$	0.723577236	τ5	300

**Table 4:** The parameters of nine linear and non-linear activation profiles for isometric contractions. $\overset{‾}{t}$ is the normalized time.

Profile Type	Equation	Profile ID	Parameters
Linear	$a\left(\bar{t};A_{0}\right)=A_{0}\bar{t}$	L1	$A_{0}=0.3$
		L2	$A_{0}=0.5$
		L3	$A_{0}=1.0$
Non-Linear	$a\left(\bar{t};k,A_{0}\right)=A_{0}\left(1-\exp(-k\bar{t})\right)$	NL1	$k=5,A_{0}=0.3$
		NL2	$k=5,A_{0}=0.5$
		NL3	$k=5,A_{0}=1.0$
		NL4	$k=10,A_{0}=0.3$
		NL5	$k=10,A_{0}=0.5$
		NL6	$k=10,A_{0}=1.0$

## Appendix A: Generalized Maxwell Visco-hyperelastic formulations

The visco-hyperelastic stress formulations are derived from the generalized maxwell model [25].

The deviatoric stresses are derived by the following equation, with superscript ‘~’, denoting hyperelastic contributions.

(22)
$$S_{ij}^{dev}(t)={\tilde{S}}_{ij}^{dev}(t)+\sum_{p=1}^{N}\;{\overset{‾}{g}}_{p}H_{p,ij}(t)$$

The deviatoric visco-hyperelastic stress variables are written as

(23)
$$H_{p,ij}(t)=\int_{0}^{t}\;exp\left[-\left(\frac{t-s}{\tau_{p}}\right)\right]\left(\frac{\partial {\tilde{S}}_{ij}^{dev}(s)}{\partial s}\right)ds$$

where ${\overset{‾}{g}}_{p}$ and $\tau_{p}(p=1\to N)$ are the N-term Prony-series deviatoric relaxation and time coefficients. To get the stress update equation in the discrete form, we evaluate the expression at time $t_{n+1}$ by decomposing the integral form,

(24)
$$H_{p,ij}\left(t_{n+1}\right)=\int_{0}^{t_{n+1}}exp\left[-\left(\frac{t_{n+1}-s}{\tau_{p}}\right)\right]\frac{d}{ds}\left({\tilde{S}}_{ij}^{dev}\left(s\right)\right)ds\;=\int_{0}^{t_{n}}exp\left[-\left(\frac{t_{n}-s}{\tau_{p}}\right)\right]\frac{d}{ds}\left({\tilde{S}}_{ij}^{dev}\left(s\right)\right)ds\;+\int_{t_{n}}^{t_{n+1}}exp\left[-\left(\frac{t_{n+1}-s}{\tau_{p}}\right)\right]\frac{d}{ds}\left({\tilde{S}}_{ij}^{dev}\left(s\right)\right)ds\;=exp\left(-\frac{\Delta t}{\tau_{p}}\right)H_{p,ij}\left(t_{n}\right)\;+\int_{t_{n}}^{t_{n+1}}exp\left[-\left(\frac{t_{n+1}-s}{\tau_{p}}\right)\right]\frac{d}{ds}\left({\tilde{S}}_{ij}^{dev}\left(s\right)\right)ds$$

where $\Delta t=t_{n+1}-t_{n}$. By using the first order derivative approximation for the stress rate inside the integral [26],

(25)
$$\int_{t_{n}}^{t_{n+1}}\;\;exp\left[-\left(\frac{t_{n+1}-s}{\tau_{p}}\right)\right]\frac{d}{ds}\left({\tilde{S}}_{ij}^{dev}(s)\right)ds\;=\frac{S_{dev,ij}^{\left(h,n+1\right)}-S_{dev,ij}^{\left(h,n\right)}}{\Delta t}\left({\left.\tau_{p}exp\left[-\left(\frac{t_{n+1}-s}{\tau_{p}}\right)\right]\right|}_{s=t_{n}}^{s=t_{n+1}}\right)\;=\left(\frac{1-exp\left(-\frac{\Delta t}{\tau_{p}}\right)}{\frac{\Delta t}{\tau_{p}}}\right)\left({\tilde{S}}_{n+1,ij}^{dev}-{\tilde{S}}_{n,ij}^{dev}\right),$$

we obtain a recursive expression for the visco-hyperelastic deviatoric internal stress variables.

(26)
$$H_{p,ij}\left(t_{n+1}\right)\approx H_{p,ij}^{n+1}=exp\left(-\frac{\Delta t}{\tau_{p}}\right)H_{p,ij}^{n}+\left(\frac{1-exp\left(-\frac{\Delta t}{\tau_{p}}\right)}{\frac{\Delta t}{\tau_{p}}}\right)\left({\tilde{S}}_{n+1,ij}^{dev}-{\tilde{S}}_{n,ij}^{dev}\right)$$

This leads to the discrete N-term Prony series stress update equation for the deviatoric stress tensor.

(27)
$$S_{ij}^{dev}\left(t_{n+1}\right)\approx S_{ij,n+1}^{dev}={\tilde{S}}_{ij,n+1}^{dev}+\sum_{p=1}^{N}\;{\overset{‾}{g}}_{p}H_{p,ij}^{n+1}$$

Similarly, the visco-hyperelastic stress update equation for the volumetric stress can be derived,

(28)
$$S_{ij,n+1}^{vol}={\tilde{S}}_{ij,n+1}^{vol}+\sum_{p=1}^{N}\;{\overset{‾}{b}}_{p}L_{p,ij}^{n+1}$$

where ${\overset{‾}{b}}_{p}(p=1\to N)$ are the N-term Prony-series volumetric relaxation coefficients and the volumetric internal stress variables are,

(29)
$$L_{p,ij}^{n+1}=exp\left(-\frac{\Delta t}{\tau_{p}}\right)L_{p,ij}^{n}+\left(\frac{1-exp\left(-\frac{\Delta t}{\tau_{p}}\right)}{\frac{\Delta t}{\tau_{p}}}\right)\left({\tilde{S}}_{n+1,ij}^{vol}-{\tilde{S}}_{n,ij}^{vol}\right)$$

## Appendix B: Visco-hyperelastic Tangent Matrix Formulation

The deviatoric tangent matrix is computed as

(30)
$$\begin{array}{r} \frac{\partial S_{ij,n+1}^{dev}}{\partial E_{kl,n+1}}=\frac{\partial {\tilde{S}}_{ij,n+1}^{dev}}{\partial E_{kl,n+1}}+\sum_{p=1}^{N}\;\;{\overset{‾}{g}}_{p}\left(\frac{1-exp\left(-\frac{\Delta t}{\tau_{p}}\right)}{\frac{\Delta t}{\tau_{p}}}\right)\frac{\partial {\tilde{S}}_{ij,n+1}^{dev}}{\partial E_{kl,n+1}} \end{array}=\left(1+\sum_{p=1}^{N}\;\;{\overset{‾}{g}}_{p}\left(\frac{1-exp\left(-\frac{\Delta t}{\tau_{p}}\right)}{\frac{\Delta t}{\tau_{p}}}\right)\right)\frac{\partial {\tilde{S}}_{ij,n+1}^{dev}}{\partial E_{kl,n+1}}$$

Similarly, the volumetric component update is written as,

(31)
$$\frac{\partial S_{ij,n+1}^{vol}}{\partial E_{kl,n+1}}=\left(1+\sum_{p=1}^{N}\;\;{\overset{‾}{b}}_{p}\left(\frac{1-exp\left(-\frac{\Delta t}{\tau_{p}}\right)}{\frac{\Delta t}{\tau_{p}}}\right)\right)\frac{\partial {\tilde{S}}_{ij,n+1}^{vol}}{\partial E_{kl,n+1}}$$

## Appendix C: Principal Strain distribution from simulation

The distributions of the maximum $\left(\epsilon_{1}\right)$ and minimum $\left(\epsilon_{2}\right)$ principal strain and maximum shear strain $\left(\gamma_{max}\right)$ are shown here.
